# Gut microbial metabolites in depression: understanding the biochemical mechanisms

**DOI:** 10.15698/mic2019.10.693

**Published:** 2019-09-27

**Authors:** Giorgia Caspani, Sidney Kennedy, Jane A. Foster, Jonathan Swann

**Affiliations:** 1 Computational Systems Medicine, Department of Surgery and Cancer, Imperial College London, UK.; 2 Centre for Mental Health and Krembil Research Centre, University Health Network, University of Toronto, Toronto, ON, CA.; 3 Mental Health Services, St. Michael's Hospital, University of Toronto, Toronto, ON, CA.; 4 Department of Psychiatry, University of Toronto, Toronto, ON, CA.; 5 Keenan Research Centre for Biomedical Science, Li Ka Shing Knowledge Institute, St. Michael's Hospital, Toronto, ON, CA.; 6 Department of Psychiatry & Behavioral Neurosciences, McMaster University, Hamilton, Ontario, Canada.

**Keywords:** microbiome, indole, tryptophan, bile acids, lactate, vitamins, mental health

## Abstract

Gastrointestinal and central function are intrinsically connected by the gut microbiota, an ecosystem that has co-evolved with the host to expand its biotransformational capabilities and interact with host physiological processes by means of its metabolic products. Abnormalities in this microbiota-gut-brain axis have emerged as a key component in the pathophysiology of depression, leading to more research attempting to understand the neuroactive potential of the products of gut microbial metabolism. This review explores the potential for the gut microbiota to contribute to depression and focuses on the role that microbially-derived molecules – neurotransmitters, short-chain fatty acids, indoles, bile acids, choline metabolites, lactate and vitamins – play in the context of emotional behavior. The future of gut-brain axis research lies is moving away from association, towards the mechanisms underlying the relationship between the gut bacteria and depressive behavior. We propose that direct and indirect mechanisms exist through which gut microbial metabolites affect depressive behavior: these include (i) direct stimulation of central receptors, (ii) peripheral stimulation of neural, endocrine, and immune mediators, and (iii) epigenetic regulation of histone acetylation and DNA methylation. Elucidating these mechanisms is essential to expand our understanding of the etiology of depression, and to develop new strategies to harness the beneficial psychotropic effects of these molecules. Overall, the review highlights the potential for dietary interventions to represent such novel therapeutic strategies for major depressive disorder.

## THE GUT MICROBIOME CONTRIBUTES TO DEPRESSIVE BEHAVIOR

**Figure 1:**
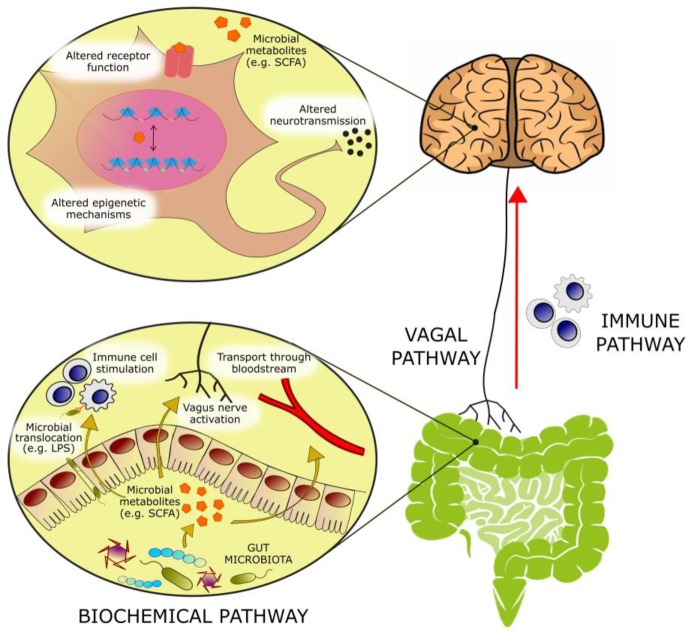
FIGURE 1: Bioactive molecules originating from microbial metabolism are thought to modulate emotional behavior through several mechanisms: **(1)** Activation of afferent vagal nerve fibers. **(2)** Stimulation of the mucosal immune system or of circulatory immune cells after translocation from the gut into the circulation. **(3)** Absorption into the bloodstream, and biochemical interaction with a number of distal organs. In the brain, such metabolites may be able to activate receptors on neurons or glia, modulate neuronal excitability, and change expression patterns by means of epigenetic mechanisms.

With an estimated three to four million different genes in the collective genomes of the gut microbiota [1] there is approximately 100 to 150 times more genetic information in the human microbiome than the human genome. Many of these genes encode proteins that perform metabolic functions and produce metabolites exclusive to the microbiome. The host encounters these metabolites in the gut, where they can exert local effects in the gastrointestinal (GI) environment or at the gut wall. Alternatively, these microbial metabolites can be absorbed, enter the systemic circulation and reach distant organs, including the brain. At these host sites, microbial metabolites can serve as ligands for host receptors with downstream effects on host gene expression and function. In addition, these microbial metabolites can integrate into host metabolic pathways altering their activity (Figure 1).

Colonization of the human gut by the microbiota is an evolutionary-driven process that impacts host physiology, for example, by priming the immune system and aiding the breakdown of otherwise indigestible fibers, and also by driving brain development and shaping behavior [2]. It is now well established that a bidirectional communication network exists between the gut and the brain, termed the gut-brain axis [3], of which the microbiota and its metabolic output are a major component. Colonization of the gut by the microbiota and central nervous system (CNS) development have extensively overlapping critical developmental windows. As a result, early-life perturbations in the maturation of the microbiota can result in deficits in neurogenesis, axonal and dendritic growth and synaptogenesis, which can negatively impact on later mental health [4]. Indeed, compared to specific pathogen-free and conventional mice, germ-free mice exhibited an exaggerated hypothalamic pituitary adrenal (HPA) axis response to restraint stress, characterized by elevated plasma adrenocorticotropic hormone (ACTH) and corticosterone as well as reduced cortical and hippocampal expression of brain-derived neurotrophic factor (BDNF) [5]. Fecal inoculation from specific pathogen-free donor mice reversed these stress-associated physiological alterations only when administered at early developmental stages. This suggests that early-life colonization by the gut microbiota is essential for the normal development of the HPA axis and of the neuroendocrine response to stress [5] and supports the notion that a limited, early critical window exists in which gut microbial stimulation shapes normal brain development [2].

Major depressive disorder (MDD) has become the leading cause of disability globally and is associated with death and suicide, more often than any other mental or physical disorder. The symptomatology of MDD includes prolonged feelings of low mood, worthlessness or guilt, anhedonia, sleep and appetite disturbances, fatigue, slowed movements and speech, and suicidal thoughts [6]. In addition to CNS abnormalities, patients with depression also exhibit alterations in metabolic, immune and endocrine systems. There is growing evidence associating the gut microbiota in the pathophysiology of depression. Several taxonomic association studies in humans have observed differences in the fecal microbiota composition of MDD patients compared to healthy subjects [7–10]. These studies identified variation in the phyla *Bacteroidetes, Proteobacteria, Actinobacteria* and *Firmicutes*, and in the genera *Enterobacteriaceae, Alistipes, Faecalibacterium, Bifidobacterium* and *Blautia*, although contradicting results were found regarding the direction of the associations detected between disease and bacterial taxa. Valles-Colomer and colleagues [11] used a module-based analytical approach of fecal metagenomes to link microbiota neuroactive capacity with depressive symptoms. This study showed a positive association between quality of life indicators and the genera *Faecalibacterium* and *Coprococcus*, as well as a negative association between the abundance of *Coprococcus spp.* and *Dialister* with depression after controlling for antidepressant use. Psychological stress can change the composition of the gut microbiota [12], and in turn, microbiota abnormalities can influence emotional behavior [13]. Germ-free rodent studies have begun to interrogate the causative role of microbiome abnormalities in the etiology of depression. Alongside the appearance of anhedonia and anxiety-like behavior, the oral gavage of fecal microbiota from MDD patients to antibiotic-treated rats induced decreased gut microbiota richness and diversity and elevated plasma kynurenine and kynurenine/tryptophan ratio [14], highlighting the potential to transfer depressive-like behavioral and physiological traits via the microbiota. Tryptophan metabolism along the serotonin (also known as 5-hydroxytryptamine or 5-HT), kynurenine and indole pathways can be influenced by the gut microbiota. The bacterial enzyme tryptophanase is responsible for the conversion of tryptophan into indole, which can give rise to a range of neuroactive signaling molecules. Additionally, tryptophan can be metabolized into 5-HT, via aromatic amino acid decarboxylase (AAAD) activity, or kynurenine by the enzymes tryptophan-2,3-dioxygenase (TDO) or the ubiquitous indoleamine-2,3-dioxygenase (IDO). Lipopolysaccharides (LPS), an inflammatory cell wall component from Gram negative bacteria, can induce the expression of IDO, increasing the conversion of tryptophan to kynurenine (reflected in the kynurenine:tryptophan ratio). The reduction in *Firmicutes* and the subsequent decrease in short-chain fatty acid synthesis observed in MDD patients has been linked to increased inflammation [15], and cytokines are also known to promote tryptophan utilization for kynurenine synthesis via IDO activity. This pathway gives rise to the neurotoxic metabolite quinolinic acid, and reduces central serotonergic availability [16]. Much of the mechanistic evidence of the involvement of the gut microbiota in depression comes from research on germ-free or on microbiota-depleted animals. Germ-free rodent models show substantial behavioral and molecular abnormalities (**Table 1**), represented by reduced anxiety and changes in central levels of several neurotransmitters, both of which could be rescued following colonization with a conventional microbiota early in life [17, 18]. Depletion of the gut microbiota by antibiotic administration was also found to induce depressive-like behaviors in adult rats, as well as altered central 5-HT availability and other depression-related physiological changes [19].

**TABLE 1.** Studies investigating the effect of a lack of microbiota on neurotransmitter systems.

**Model**	**Species or strain**	**Behavioral outcomes**	**Molecular mechanisms**	**Reference**
GF	Adult male BALB/c mice (7–9 weeks)	-	Biologically inactive and conjugated form of colonic norepinephrine and dopamine in GF mice (compared to the biologically active, free form in conventional mice); reduced intestinal norepinephrine and dopamine rescued by microbiota recolonisation	[45]
GF	Male Swiss Webster mice (8–10 weeks)	-	altered blood concentrations of indole derivatives (including ↑ tryptophan and ↓5-HT), phenyl derivatives (including ↑ tyrosine) and other metabolites in GF compared to conventional mice	[32]
GF	Male BALB/c mice (7 weeks)	-	Altered cerebral metabolome (including ↓ tryptophan and tyrosine, but ↑ dopamine and N-acetylaspartatic acid) of germ-free mice compared to Ex-GF mice, which were inoculated with suspension of feces from SPF mice; reduced GABA in faeces and blood (but not in brain) rescued by microbiota recolonisation	[46]
GF	Male and female Swiss Webster mice	↑ anxiety phenotype normalised by conventionalisation	↓ immune response and ↑ HPA axis reactivity in GF mice; ↓ BDNF expression in hippocampus; ↑ hippocampal 5-HT and 5-HIAA in males only; ↑ plasma tryptophan availability and ↓ kynurenine:tryptophan ratio in males (restored by colonisation); ↑ hippocampal 5-HT and 5-HIAA not normalised by conventionalisation	[49]
GF	Male mice (8-10 week)	↓ anxiety-like behavior	↑norepinephrine, Dopamine, and 5-HT turnover in the Striatum; Altered Expression of Synaptic Plasticity-Related Genes; Colonization of GF Mice Reduces Protein Expression of Synaptophysin and PSD-95 in Striatum	[2]
GF	C57Bl/6J mice	-	↓ circulatory and faecal (colonic ECs) 5-HT in GF compared to SPF mice; colonisation of GF mice with SPF microbiota restores serotonergic abnormalities, elevates TPH1 expression and decreases SLC6A4 expression.	[48]
GF	BALB/c mice	-	Altered intestinal concentration of several metabolites (including ↓ GABA in GF compared to colonised mice)	[53]
GF	Male Swiss Webster mice (12–14 weeks)	-	Altered levels of microbial metabolites in serum of GF compared to conventional mice, including ↓serum concentrations of dopamine and tyramine and of trans - 2-aminomethylcyclopropanecarboxylic acid, a cyclopropane analog of GABA	[52]

**5-HIAA**: 5-Hydroxyindoleacetic Acid; **5-HT**: 5-Hydroxytryptamine; **BDNF**: Brain-Derived Neurotrophic Factor; **GABA**: Gamma-Aminobutyric Acid; **GF**: Germ-Free; **HPA**: Hypothalamic-Pituitary-Adrenal; **PSD-95**: Postsynaptic Density Protein 95; **SLC6A4**: Serotonin Transporter; **SPF**: Specific Pathogen Free; **TPH-1**: Tryptophan Hydroxylase 1.

## PATHWAYS OF MICROBIOTA-GUT-BRAIN-COMMUNICATION

The gut microbiota and its metabolic products can affect central physiological and pathological processes through several proposed mechanisms. Neural communication between the gut and the brain is mainly mediated by intestinal afferent fibers of the vagus nerve. Vagal stimulation by the gut microbiota or its metabolites is relayed to the nucleus tractus solitarius, and then transmitted to the thalamus, hypothalamus, locus coeruleus, amygdala and periaqueductal grey [3]. Electrical stimulation of the vagus nerve by the gut microbiota can alter the concentrations of neurotransmitters like 5-HT, γ-aminobutyric acid (GABA) and glutamate in the brain of both rodents and humans [20]. Additionally, rodent studies have shown that the anxiety and depressive phenotype that is normally induced by an immune challenge can be prevented by vagotomy [21, 22], supporting the role played by the vagus nerve in stress reactivity and emotional regulation.

The immune system represents a major component of gut-to-brain communication. While central immune cells and low levels of inflammatory mediators exert a variety of physiological roles in the brain (ranging from sleep to memory formation), sustained neuroinflammation has deleterious effects on brain function and has been associated with a variety of neuropsychiatric disorders [23]. The gut microbiota has important roles in shaping immune function throughout life. In early life, it directs normal development of central immune cells, like microglia and astrocytes [24]; in adulthood, it sets a chronic physiological state of low-grade inflammation [25], as the bacterial antigens present in the intestinal tract stimulate cytokine release by intestinal macrophages and T cells [26]. Peptidoglycans derived from bacterial cell walls have been measured in the brain, where they activate central pattern-recognition receptors to stimulate the innate immune system and alter behavior [27]. These observations are consistent with a role for immune molecules in the CNS independent of infection or immune stimulation, but actually a component of normal healthy brain function.

The gut microbiota is also central to brain function in the context of an immune challenge. LPS can trigger the release of the cytokine IL-18 [28]. Parenteral administration of LPS to healthy individuals induced immune system activation accompanied with mild depressive and cognitive symptoms [29]. Significantly, LPS translocation into the brain is suggested to be under the control of propionate, a gut microbial metabolite that modulates blood-brain barrier (BBB) permeability [30]. Pro-inflammatory cytokines in the GI tract can also modulate central stress circuitry by stimulating the vagus nerve and activating the HPA axis [31]. Stress and dietary patterns such as the Western diet can contribute to neuroinflammation by increasing the permeability of the intestinal wall, a pathological state referred to as “leaky gut”. This allows the translocation of bacteria and LPS from the intestinal lumen into the bloodstream and the CNS [25].

Finally, direct biochemical signaling can take place by means of bioactive molecules of bacterial origin. Extensive studies in germ-free and antibiotic-treated rodents have highlighted the diverse biochemical output of the microbiome. This diversity is a product of the chemically heterogeneous substrate entering the gut from both the diet and host secretions as well as from the expansive metabolic repertoire of the microbiome [32]. Metabolites produced in the gut by the bacterial fermentation of dietary components can be absorbed in the bloodstream and interact with enzymes and receptors expressed by the host, contributing to both physiological and pathological processes in the host [33]. To date, evidence suggests that microbiota-derived acetate can act remotely to influence neural function [34]. Neurotransmitters, short-chain fatty acids (SCFAs), bile acids, choline metabolites, lactate and vitamins are products of gut microbial metabolism that can directly or indirectly influence central processes and, when dysregulated, contribute to neuropathology [25]. This review will focus on the potential of these metabolite classes to alter biochemical processes underlying gut-to-brain communication, and describe the role played by these microbial metabolites in the pathophysiology of depression.

## NEUROACTIVE MICROBIAL METABOLITES AND THEIR ROLE IN DEPRESSION

### Neurotransmitters

The majority of central neurotransmitters are also present in the GI tract, where they exert local effects ranging from modulating gut motility and secretion to cell signaling [35, 36]. Members of the gut microbiota can synthesize neurotransmitters: *Lactobacilli* and *Bifidobacteria* produce GABA [37–41]; *Escherichia coli (E. coli)* produce 5-HT and dopamine [42, 43]; *Lactobacilli* produce acetylcholine [44], and many more microbial taxa contribute to the synthesis and release of other molecules with neuroactive properties. In fact, gut microbiota absence is associated with significant reductions in intestinal levels of neurotransmitters like norepinephrine [45], 5-HT [32], and GABA [46]. While recolonization can re-establish normal neurotransmitter concentrations, it is not clear if this restoration of neurotransmission is due to bacterially derived products or due to stimulation of neurotransmitter producing host intestinal cells [47]. An example of the latter is 5-HT, whose intestinal concentrations are maintained by enterochromaffin cells, which express the enzyme tryptophan hydroxylase upon stimulation by gut metabolites such as SCFAs and secondary bile acids [48].

It is now established that peripheral production of neurotransmitters by the gut microbiome may alter brain chemistry and influence behavior (**Table 2**). While there is no evidence that gut-derived neurotransmitters reach the brain, these compounds may influence CNS signaling by co-feeding of other commensal bacteria and modulation of local host gut physiology upon absorption into the bloodstream. For example, Clarke *et al.* [49] showed that male germ-free mice exhibit anxiety-like behaviors as well as altered neurotransmitter (5-HT and 5-hydroxyindole acetic acid (5-HIAA)) abundance in the hippocampus. These central alterations were accompanied by an elevation in plasma tryptophan concentrations, suggesting that the peripheral tryptophan metabolism is influenced by microbiota, that also influence CNS neurotransmitter systems. While abnormal anxiety behavior was normalized by conventionalization in adulthood, the neurochemical imbalances in male germ-free mice persisted, indicating the profound effects of the gut microbiota on the development of neurotransmission [49]. The concentrations of dopamine and norepinephrine were also increased in the brains of germ-free mice in a separate study [17]. Additionally, a study chronically administering *L. rhamnosus* to mice reported changes in GABA_A_ and GABA_B_ receptor expression as well as in the levels of brain activity, accompanied by a reduction in anxiety and depression-like symptoms [50]. Similarly, the GABA-producing *L. brevis FPA3709* had an antidepressant effect when administered to rats [51]. Lower circulating concentrations of 5-HT [32, 48], dopamine [52] and GABA [53] have been observed in germ-free mice. This finding suggests that the gut microbiota may modulate neurotransmission via the bloodstream. Although enhancing 5-HT production in the gut does not result in an increase in central concentrations [47]****, central concentrations of 5-HT can be enhanced by increasing the concentrations of its precursor tryptophan in the GI tract [16, 54]. These findings have an important relevance in the context of depression, as they demonstrate the possibility of modulating central serotonergic neurotransmission through non-invasive interventions that target the gut microbiome.

**TABLE 2.** Studies investigating the effects of treatment with microbial cultures on neurotransmission and behavior.

**Treatment**	**Species or strain**	**Behavioral outcomes**	**Molecular mechanisms**	**Reference**
*L. rhamnosus* (109 cfu daily for 28 days)	Adult male BALB/c mice (10–11 weeks)	↓ anxiety and depressive-like behavior in OFT, SIH, EPM, fear conditioning (contextual and cued), and FST after L. rhamnosus chronic adnimistration; vagotomy prevented the anxiolytic and antidepressant effects of *L. rhamnosus*	Changes in expression of GABAAα2 and GABAB1b mRNA after L. rhamnosus chronic administration; vagotomy alone was sufficient to increase GABAAα2 mRNA in the hippocampus but prevented further changes induced by *L. rhamnosus*	[55]
*B. infantis* (daily for 14 days)	Sprague-Dawley rats	no effect on depressive-like behavior	↓ inflammatory markers (IFN-γ, TNF-α and IL-6 cytokines); ↑ plasma tryptophan and kynurenic acid; ↓concentrations of 5-HIAA (frontal cortex) and DOPAC (amygdaloid cortex)	[58]
*L. rhamnosus* (1 × 109 cfu daily for 4 weeks)	male BALB/c mice	↓ in anxiety and depression-related behaviors	↑ glutamate + glutamine and ↑ total N-acetyl aspartate + N-acetyl aspartyl glutamic acid at 2 weeks; ↑ GABA at 4 weeks	[50]
*L. brevis* FPA 3709 (48-h fermented black soybean milk at a dosage of 35 mg/kg b.w. including 2.5 mg GABA/kg b.w. for 28 days)	Male Sprague-Dawley rats	↓ in depressive behavior in FST comparable to the effect of fluoxetine	-	[51]

**5-HIAA**: 5-Hydroxyindoleacetic Acid; **cfu**: Colony-Forming Unit; **DOPAC**: 3,4-Dihydroxyphenylacetic Acid; **FST**: Forced Swim Test; **EPM**: Elevated Plus Maze; **GABA**: Gamma-Aminobutyric Acid; **IFN-γ**: Interferon Gamma; **IL-6**: Interleukin-6; **OFT**; Open Field Test; **SIH**: Stress-Induced Hyperthermia; **TNF-α**: Tumor Necrosis Factor Alpha.

Microbial metabolites can also have an impact on central neurotransmission by activating afferent nerve fibers. The involvement of the vagus nerve in gut-brain communication was demonstrated by Bravo *et al.* [55]. This work showed that administration of probiotics like *L. rhamnosus* had anxiolytic and antidepressant effects and induced significant changes in GABA receptor expression in the brain of normal, but not vagotomized, mice [55]. Neurotransmitters produced in the gut may also influence brain function through the modulation of the immune system. There have been reports of 5-HT activating cells of the immune system [56], and of GABA dampening intestinal inflammation [57]. Upon chronic administration of the probiotic *B. infantis*, naïve rats displayed an attenuation of inflammatory markers such as interferon-γ (IFN-γ), tumor necrosis factor-α (TNF-α) and interleukin-6 (IL-6) [58]. Since a concomitant increase in circulating tryptophan and kynurenic acid and decrease in central 5-HIAA and 3,4-dihydroxyphenylacetic acid (DOPAC) were described, the dampening of the inflammatory response may be ascribed to a change in neurotransmitter production and availability [58]. Alternatively, neurotransmitters produced by the gut microbiota can inhibit cytokine production through local stimulation of the vagus nerve [59].

These studies suggest that neurotransmitters produced, either directly or indirectly, by gut bacteria may influence emotional behavior by binding specific receptors in the CNS, or peripheral receptors on neural or immune cells. A wider range of bacterially-derived, bioactive, transmitter-like molecules may exist whose effects on depressive symptoms have not been investigated to the same extent as classic neurotransmitters. These molecules include histamine, gasotransmitters (*e.g.* nitric oxide, ammonia), neuropeptides, endocannabinoids, steroids [60], and it is likely that more will be identified in the future. This communication between bacterial and host metabolism of neurotransmitters is bidirectional in nature: in addition to synthesizing neurotransmitters that are able to alter host physiology, gut microbes can also respond to neurotransmitters produced by the host, which influence bacterial growth and development [61].

### SCFAs

SCFAs are small organic compounds produced in the cecum and colon by anaerobic fermentation of predominantly indigestible dietary carbohydrates that cross-feed other bacteria and are readily absorbed in the large bowel [62]. SCFAs are involved in digestive, immune and central function, although different accounts on their impact on behavior exist. Administration of the three most abundant SCFAs (acetate, butyrate and propionate) was shown to alleviate symptoms of depression in mice [63]. In support of their involvement with the etiology of depression, a depletion of butyrate, acetate and propionate was reported in MDD patients [8, 10, 64, 65], and a high abundance of butyrate-producing bacteria, like *Faecalibacterium* and *Coprococcus spp.*, was detected in subjects with higher quality of life indicators [11]. The genera *Faecalibacterium* and *Coprococcus* are Gram-positive, anaerobic bacteria which ferment dietary fibers to produce SCFAs. *Faecalibacteria* are one of the most abundant gut microbial genera, with important immunological functions and clinical relevance for a variety of diseases, including MDD [8].

SCFAs are able to bind and activate the G protein-coupled receptors GPR43 (free fatty acid receptor 2 (FFAR2)) and GPR41 (FFAR3), as well as the less common CPR164 and GPR109a (also known as OR51E1 and HCAR2 respectively) [66]. These receptors are ubiquitously expressed by several organs in the body, including enteroendocrine cells, adipocytes, immune cells and neurons [66], suggesting that SCFAs may alter behavior by direct stimulation of neural pathways, or through the indirect central effect of neuroendocrine and immune activation.

Locally, SCFAs promote gut health by modulating energy regulation, glucose metabolism and lipid homeostasis [67] and regulate intestinal barrier integrity by enhancing the expression of tight junctions (particularly butyrate, see [68]). By binding to FFAR2, SCFAs control feeding behavior by stimulating the production of the anorexigenic hormones glucagon-like peptide-1 (GLP-1) and peptide YY (PYY) by enteroendocrine cells [69–71], and of leptin by adipocytes [72]. As previously stated, SCFAs also contribute to the synthesis and release of peripheral neurotransmitters (like 5-HT and acetylcholine) by enterochromaffin cells, in a process that is thought to be mediated by OR51E1 [73], and norepinephrine by sympathetic neurons, via stimulation of FFAR2 and FFAR3 [74]. Recent work has demonstrated the presence of FFAR3 in the mouse vagal ganglia [75], suggesting a role for SCFAs in establishing visceral reflexes. The ability of SCFAs to activate vagal fibers and induce activity in the hypothalamus has been implicated as the neural basis of their central anorexigenic effect [76]. In addition to their local action in the gut and in the peripheral nervous system, SCFAs can act directly on central receptors due to their ability to diffuse passively or actively (via monocarboxylate transporters) across the BBB [77, 78]. SCFAs like acetate can directly modulate appetite by binding to and activating receptors in the hypothalamus [34]. Interestingly, appetite suppression by propionate involves the attenuation of neural activity in regions of the brain reward system (i.e. caudate and nucleus accumbens) [79], a circuitry that is also dysfunctional in patients with depression [80]. Since no change in circulatory concentrations of PYY or GLP-1 were observed, it is likely that signaling via the vagus nerve or central receptors is responsible for the central effects of propionate. In addition, *in vitro* studies show both propionate and butyrate, but not acetate, can modulate the permeability of the BBB, protecting against the increased permeability caused by LPS [30].

Binding of SCFAs to FFAR2, FFAR3, GPR109a and Olfr78 receptors expressed by immune cells contributes to the development and function of the immune system [81]. For example, microglia abnormalities in germ-free mice can be reversed by SCFA administration in a FFAR2-dependent manner [82]. The observation that SCFAs generally dampen inflammation [83, 84] suggests that the antidepressant effects of SCFAs may be partly accounted for by their anti-inflammatory properties. However, while butyrate was shown to suppress neuroinflammation by acting on microglial GPR109a receptors [68], propionic acid was shown to activate microglia and induce reactive astrogliosis in rats [85] and to promote immune cell recruitment in a FFAR3-mediated pathway [86]. These observations suggest a complex relationship between SCFAs and immune function. Moreover, while central butyrate promotes neurogenesis and angiogenesis [87, 88] and contributes to tight junction expression and BBB structural integrity [68], intraventricular infusions of propionate contributed to mitochondrial dysfunction and oxidative stress by inducing lipid peroxidation, protein carbonylation and metabolic alterations in the rat brain [85, 89, 90]. However, it must be noted that many of these preclinical studies used supraphysiological doses of propionate and that, while intraventricular injections elicited a strong effect on the brain, such changes do not occur if SCFAs were administered peripherally.

SCFAs are strong epigenetic modulators that can control the accessibility of genetic material for DNA methylation and inhibition of histone deacetylation. A rodent study revealed the DNA methylation properties of sodium butyrate, the salt form of butyric acid [91]. This mechanism is dependent on ten-eleven translocation (TET) proteins, which catalyze the hydroxylation of cytosine residue (5mC) into 5-hydroxymethylcytosine (5hmC). 5hmC can then mediate active DNA demethylation. While depressed mice exhibited low levels of the TET methylcytosine dioxygenase 1 (TET1), mice treated with sodium butyrate showed a normalization in 5-hydroxymethylation levels by TET1, resulting in BDNF gene overexpression [91]. Depression is often characterized by altered histone deacetylase (HDAC) activity, and several studies have demonstrated the epigenetic potential of different antidepressant medications [92]. Butyrate has been identified as a HDAC inhibitor for HDAC1, HDAC2 and HDAC7 [93], and its systemic administration induced histone acetylation in the hippocampus and frontal cortex in mice [94]. The beneficial effect of sodium butyrate on mood was shown in rodent models of depression either alone [95–97] or in conjunction with antidepressant drugs [94, 98]. For example, repeated injections of sodium butyrate reversed the LPS-induced activation of microglia and depressed mood in mice [99]. This antidepressant effect was mediated by the acetylation of hippocampal histones H3 and H4, which reduced the expression of Iba1, a marker of microglia activation [99]. Alternatively, Sun *et al.* [100] found that the beneficial effects of sodium butyrate on depressive behavior were mediated by an increase in 5-HT concentrations, reversal of hippocampal neuronal abnormalities, increased BDNF expression, and an upregulation of tight junction expression at the BBB [100]. In line with these findings, sodium butyrate was reported to promote the expression of dopamine, adrenaline, and other neurotransmitter genes in a rat pheochromocytoma cell line [101]. Other investigations demonstrated that further effects of HDAC inhibition by butyrate included a reduction in neuroinflammation through modulation of microglia activation [102] and an enhancement in *N-*methyl-D-aspartate (NMDA) receptor activity [103].

The SCFA propionate also acts as a HDAC inhibitor [104], and intrarectal administration of sodium propionate was shown to improve despair behavior in rats [105]. The antidepressant effect of propionate was accompanied by an increase in norepinephrine, dopamine, tryptophan, 5-HIAA and 3-hydroxyanthranilic acid (3-HAA) in the prefrontal cortex, although no change was detected in 5-HT and 3-hydroxykynurenine (3-HK). The known ability of propionate (shared with butyrate) to promote dopamine and norepinephrine synthesis by enhancing the transcription of the tyrosine hydroxylase gene [101], may be the mechanism underlying these molecular and behavioral effects. Both butyrate and propionate may also contribute to dopaminergic function by inhibiting the expression of dopamine-β-hydroxylase, which catalyzes the conversion of dopamine into norepinephrine. Thus, the opposite effects of SCFAs on behavior may be explained by their action via independent mechanisms: for example, Li *et al.* [105] found that while butyrate modulated the expression of 5-HT (with slow-onset but long-term antidepressant action), propionate altered the expression of norepinephrine (with fast-acting, but short-term antidepressant action). However, propionate is also able to modulate serotonergic function by increasing the expression of tryptophan hydroxylase (TPH) [101], responsible for the conversion of tryptophan to 5-HT. This finding is significant for unveiling the link between neuroinflammation and neurotransmitter production, as an increased TPH turnover induces an accumulation of kynurenine and neurotoxic metabolites like 3-HK [105]. Since altered tryptophan–kynurenine metabolism is characteristic of depression [106], this observation suggests that serotonergic function may be linked to anti-inflammatory mechanisms. Indeed, oral administration of propionate was shown to result in a decrease in the neurotransmitters GABA, 5-HT, and dopamine, as well as in a range of biomolecular alterations which included increased oxidative stress (indicated by lipid peroxidation), altered energy metabolism, and higher pro-inflammatory markers like IL-6, TNF-α, IFN-γ, heat shock protein 70 and caspase 3 [107]. Several additional studies suggested that modulation of mood by SCFAs can occur via mechanisms involving the immune system, but the findings are contradictory. While butyrate has established anti-inflammatory effects including the inhibition of pro-inflammatory gene expression [108–110], propionate has been reported to have both anti- [111] and pro-inflammatory properties [112–114].

Despite this evidence (**Table 3**), results supporting the antidepressant potential of SCFAs are not consistent enough to be translated into medical practice. For example, cecal isobutyrate is reduced in response to administration of probiotics with antidepressant efficacy [115], and some studies have failed to detect significant abnormalities in the abundance of butyrate in MDD patients [116, 117] or animal models of depression [105] compared to controls. Such discrepancies may be partly due to the highly volatile nature of SCFA and to their sensitivity to the conditions of storage and tissue extraction [118], which can affect quantification and hinder comparable results across studies. In addition, controversies exist regarding the appropriate control for studies that administer SCFA in the form of salt. Although the ideal control for this experimental model should be sodium matched, some behavioral and/or physiological effects cannot be excluded [63], especially in the light of recent findings showing that a diet high in salt alters gut microbiota composition and reduces butyrate production [119]. As for propionate, its dysregulation in animal models of depression has been consistently demonstrated [64, 105], but its neurotoxic effects and the behavioral deficits elicited at excessive doses imply that more in-depth knowledge of the underlying mechanisms are required before a targeted intervention can be developed.

**TABLE 3.** Studies investigating the effects of SCFAs on depressive-like behavior.

**Treatment**	**Species or strain**	**Model**	**Behavioral outcomes**	**Molecular mechanisms**	**Reference**
Sodium butyrate (100 mg/kg or 1.2 g/kg; ip; 1 or 21 days)	129SvEv x C57BI/6 mice (F1 crosses)	-	↑ immobility time and ↑ latency to consume peanut butter chips in the novel environment after acute treatment with SB100; no effect of chronic treatment	↑ acH4/H3 and acH3/H3 protein in HP after acute SB 100 and/or 1.2 treatment, respectively;↓ acH4/H3 (no changes in acH3/H3) in HP after chronic SB100 administration	[95]
Sodium butyrate (1.2 g/kg ip; 1 or 7 days)	Sprague-Dawley rats	-	↓ immobility time in rats after repeated (but not acute) SB administration; no changes in OFT	↑ Ttr and ↓ Slc8a3, Casr, Htr2a, Tcf12 and no changes in Sin3a, Gnrhr, Crhr2, Bdnf, Slc8a2 gene expression in hippocampus after repeated SB treatment; ↑ levels of acH4-associated DNA at the Ttr promotor region in HP of rats repeated treated with SB; ↑ Ttr and no changes in acH3/H3, acH4/H4 protein level in HP after repeated SB administration	[97]
Sodium butyrate (0.3 g/kg ot 0.6 g/kg)	ICR mice	CRS	↓ anhedonia, time spent in dark and immobility time after SB0.6 administration in CRS-treated mice	SB0.6 reverses CRS-induced decrease in acH3 level in HP	[96]
Fluoxetine (10 mg/kg; oral) + Sodium butyrate (300 mg/kg; ip) for 21 days	Sprague-Dawley rats (2 months)	-	↓ time spent in social grooming and frequency of pouncing and ↑ immobility time and immobility events in PNFlx rats; ↑ latency to approach center and ↓ time spent in the center and path length in the center in PNFlx animals; postnatal treatment with SB and adult fluoxetine (AFlx) treatment prevented the PNFlx-evoked behavioral changes	↑ Hdac4, Ppp2r2b, Gal, Dcx, Kcnh2, Grm8, ElkI and ↓mTOR, Gnai1, Prkcc, HcnI, Notch3 and Avpr2 mRNA levels in HP of PNFlx rats;co-administration of SB prevented the PNFlx-evoked dysregulation of Hdac4 and mTOR, but not Gnai1, Prkcc and HcnI in HP; AFlx administration did no alter hippocampal expression of Hdac4, mTOR, Gnai1, HcnIand Prkcc; ↑ acetylation of H3 and H4 at the Hdac4 promoter and ↑ HDAC4 enrichment in Gnai1 and mTOR promoter in HP of PNFlx rats and normalization after adult fluoxetine treatment; ↓ mTOR protein level in HP of PNFlx rats and no changes after AFlx treatment	[98]
Sodium butyrate (0.4 g/kg; ip) twice a day for 23 days	Male FRL and FSL rats (3 months)	-	chronic NaB-treatment rescued the FSL depression-like phenotype; ↓ immobility time	The FSL-NaB group exhibited ↑ Tet1 mRNA (and protein); ↓ Dnmt1 mRNA (but not protein) levels in the FSL-NaB group; ↓ 5hmC levels at the Bdnf P4 locus; hypermethylation of Bdnf P4 compared to FRL-Veh; the NaB-dependent increase in 5hmC levels in Bdnf P4 of FSL was associated with DNA hypomethylation at the same locus; NaB-dependent increase in TET1 and 5hmC levels in the FSL group was associated with a Bdnf P4 overexpression	[91]
Sodium butyrate (500 mg/kg, i.p.) twice a day for 7 days	Wistar rats (2 months)	maternal deprivation or CMS	↓depressive-like behavior in FST	↑ tricarboxylic acid cycle anzyme (succinate dehydrogenase and malate dehydrogenase) and mitochondrial chain complexes (I, II, II-III and IV) activity in the striatum	[103]
Propionate	Sprague-Dawley rats	CUMS	Improved performance at the SPT and OFT; short-term antidepressant-like effects.	Restored plasma levels of propionic acid; ↑NE, DA, TRP, 5-HIAA, and 3-HAA in the PFC (no effects on 5-HT and 3-HK) were not; ↓ turnover of TRP to KYN (calculated as KYN/TRP) and ↓turnover of DA to HVA (calculated as HVA/DA); ↑ abundance of DOPAC and 3-MT, but no change in HVA; no effect on turnover of 5-HT to 5-HIAA (calculated as 5-HIAA/5-HT); ↑turnover of KYN to 3-HK	[105]
Sodium butyrate (200 mg/kg) or fluoxetine (20 mg/kg)	Male C57BL/B6 mice	CUMS	↑ sucrose intake in SPT; ↑ locomotor activities in OFT; decreases immobility time in TST and FST	decreases histological abnormalities in hippocampal neurons; ↑ BDNF expression; ↑ Occludin and ZO-1 protein levels	[100]
Sodium butyrate (1.2 g/kg or 0.2 g/kg, i.p.); fluoxetine (10 mg/kg, i.p.)+ SB (0.6 mg/kg, i.p.) acutely of chronically (28 days)	male and female C57BL/6J mice (9-22 weeks)	-	improved performance at the TST	↑ histone acetylation in the brain; ↑ BDNF in mouse frontal cortex	[94]

**3-HAA**: 3-Hydroxyanthranilic Acid; **3-HK**: 3-Hydroxyanthranilic Acid; **3-MT**: 3-Methoxytyramine; **5-HIAA**: 5-Hydroxyindoleacetic Acid; **5hmc**: 5-Hydroxymethylcytosine; **5-HT**: 5-Hydroxytryptamine; **Ach4/H3**: Acetylated Histone H3/4; **Avpr2**: Arginine Vasopressin Receptor 2; **Casr**: Calcium-Sensing Receptor; **CMS**: Chronic Mild Stress; **Crhr2**: Corticotropin Releasing Hormone Receptor 2; **CRS**: Chronic Restraint Stress; **CUMS**: Chronic Unpredictable Mild Stress; **DA**: Dopamine; **Dcx**: Dublecortin; **Dnmt1**: DNA (Cytosine-5)-Methyltransferase 1; **DOPAC**: 3,4-Dihydroxyphenylacetic Acid; **Elkl**: ETS Domain-Containing Protein; **FRL**: Flinders Sensitive Line; **FSL**: Flinders Resistant Line; **FST**: Forced Swim Test; **Gal**: Galanin; **Gnai1**: G Protein Subunit Alpha I1; **Gnrhr**: Gonadotropin Releasing Hormone Receptor; **Grm8**: Glutamate Metabotropic Receptor 8; **Hcnl**: Hyperpolarization-Activated Cyclic Nucleotide-Gated Channel 1; **Hdac4**: Histone Deacetylase 4; **Htr2a**: 5-Hydroxytryptamine Receptor 2A; **HVA**: Homovanillic Acid; **Kcnh2**: Potassium Voltage-Gated Channel Subfamily H Member 2; **KYN**: Kynurenine; **Mtor**: Mammalian Target of Rapamycin; **NE**: Norepinephrine; **Notch3**: Neurogenic Locus Notch Homolog Protein 3; **OFT**: Open Field Test; **PFC**: Prefrontal Cortex; **Ppp2r2b**: Protein Phosphatase 2 Regulatory Subunit Beta; **Prkcc**: Protein Kinase C Gamma; **Sin3a**: SIN3 Transcription Regulator Family Member A; **Slc8a3**: Solute Carrier Family 8 Member A3; **SPT**: Sucrose Preference Test; **Tcf12**: Transcription Factor 7-Like 2; **TET1**: Ten-Eleven Translocation 1; **TRP**: Tryptophan; **TST**: Tail Suspention Test; **Ttr**: Transthyretin; **ZO-1**: Zonula Occludens-1.

For example, there is still a lack of consensus regarding the mode of action and receptor specificity of SCFAs. In addition, it remains unclear how well the microbial production of SCFAs in the gut parallels CNS availability. It is known that lumen concentrations of SCFAs are highly variable among individuals, and can range between 20-140 mM depending (among other factors) on fiber content of the diet, microbiota composition, rate of absorption and site of measurement in the gut [120, 121]. Absorbed by colonocytes, SCFAs are transported to the liver and then enter the systemic circulation in much lower concentrations (0.1–10 mM) [122, 123]. Although it remains unclear how well the microbial production of SCFAs in the gut relates to CNS availability, rodent studies have shown that ~3% of acetate administered intravenously reaches the CNS [34], suggesting that only a small proportion of the SCFAs absorbed from the gut reaches the brain. Increasing bacterial production of SCFAs by means of higher fiber intake (reviewed in [124, 125]) and pre- or probiotics use [126, 127] have been shown to effectively enhance the concentrations of SCFAs in the gut. The question remains as to whether direct SCFA supplementation is more effective than strategies targeting the gut microbiota. While direct supplementation with SCFAs may overcome problems related to competition of probiotic strains with resident bacterial strains, care has to be taken to elucidate the effects of SCFA depending on whether it is administered acutely (i.e. via supplementation) or chronically (i.e. via microbial production). Thus, the best strategy to implement the known beneficial effects of SCFAs on mood has still to be elucidated.

### Tryptophan metabolites

Tryptophan is an essential amino acid involved in protein synthesis [128]. Its metabolic breakdown by host (TDO and IDO) and bacterial enzymes (tryptophanase) give rise to neuroactive molecules with established mood-modulating properties, including 5-HT, kynurenine and indole. It is well-established that dietary intake of tryptophan can modulate central concentrations of 5-HT in humans [129, 130], and that tryptophan depletion exacerbates depressive symptoms in healthy individual at risk for depression [131, 132], as well as remitted [133–135] and currently depressed patients [136, 137]. However, less than 5% of tryptophan is converted into 5-HT along the methoxyindoles pathway by the enzyme tryptophan hydroxylase; the remaining 95% is metabolized along the kynurenine pathway by the enzymes TDO and IDO. Kynurenine can be further metabolized into kynurenic acid (KYNA) or, alternatively, into quinolinic and picolinic acids via the nicotinamide adenine dinucleotide (NAD) pathway. KYNA is an NMDA and α7 nicotinic acetylcholine receptor antagonist; quinolinic and picolinic acids are NMDA agonists with neurotoxic and pro-depressant effects [138]. Over-stimulation of the kynurenine pathway leads to increased lipid peroxidation and inflammation, due to quinolinic and picolinic acids and free radical generation (3-hydroxykynurenine and 3-hydroxyanthranilic acid) [139, 140]. Conversely, production of stress hormones (i.e. cortisol) and pro-inflammatory cytokines (i.e. interferons, TNF-α, interleukins) stimulate TDO and IDO formation respectively, enhancing kynurenine output at the expenses of 5-HT synthesis. In turn, the weakening of the inhibitory feedback of 5-HT on cortisol production contributes to the worsening of this cycle [141]. Therefore, disturbances in tryptophan metabolism (i.e. the shunt of tryptophan from 5-HT to kynurenine synthesis) may be partly responsible for the mood, cognitive and sleep disturbances typical of depression [141].

The mechanisms that control the uptake of tryptophan into the brain are not fully understood: these include the proportion of circulatory tryptophan that is bound to albumin (which is unable to cross the BBB), as well as the competition with other neutral amino acids for its transport through the BBB [142], but other factors are likely to be involved. Studies on germ-free animals have demonstrated the role of the microbiome in mediating the behavioral effects of tryptophan metabolism, suggesting a potential additional mechanism. Upon colonization of these animals with tryptophan-metabolizing bacteria, a decrease in tryptophan and an increase in hippocampal 5-HT concentrations was noted, accompanied by reduced anxiety-like behaviors [2, 49]. Studies have shown that the metabolic activity of the gut microbiota on dietary tryptophan produces biologically active signaling molecules, such as indole and its derivatives. Indole is an aromatic amino acid produced through the microbial metabolism of tryptophan by bacteria expressing the enzyme tryptophanase (e.g*. E. coli* [143] and other strains [144]). In microbial communities, indole is used as a quorum-sensing signal to coordinate collective behaviors like spore formation, plasmid stability and drug resistance [144]. Moreover, it plays an important role in gut physiology as it stimulates enteroendocrine L cells to secrete GLP-1 [145] and regulates gut barrier permeability [146]. In addition, oxindole and isatin (2,3-dioxoindole), products of indole oxidation and conjugation respectively, have been described as neuroactive signaling molecules able to modulate motor function and emotional behavior. Oxindole is a strong inhibitor of motor activity, and it is known to result in loss of the righting reflex, hypotension, and reversible coma [147]. Isatin increases water intake and decreases food intake. A rodent study using antagonists selective to specific receptors highlighted the possibility of these effects being mediated by the 5-HT_3_ receptor and the dopamine D_2_ receptor [148]. The action of isatin on 5-HT_3_ and atrial natriuretic peptide (ANP) receptors may also be responsible for the negative effect of this compound on memory formation [149]. Additionally, isatin is an endogenous monoamine oxidase (MAO) B inhibitor and a benzodiazepine receptor antagonist. As such, it has an established anxiogenic profile in both mice and rats [150, 151], and in turn, its production is drastically increased in conditions of stress. However, it is important to state that modifications in the chemical structure of indole and derivatives have been reported to drastically change the behavioral properties of these compounds, and even confer some antidepressant actions [152].

Based on research studies investigating the behavioral effects of indole and its metabolites, several pathways may mediate the neuroactive potential of indoles (**Table 4**). Enhanced tryptophan catabolism into indoles may mimic the reversible effect of a tryptophan-deficient diet, which is also associated with reduced 5-HT availability and increased neuroinflammation [153]. Other mechanisms may include direct effects of indole metabolites on central receptors, activation of the vagus nerve by gut bacteria or their metabolites, and stimulation of a neuroinflammatory state. A study by Jaglin *et al.* [154] showed how the effects of indole on physiology and behavior were mediated by different pathways depending on whether they were administered chronically or acutely. Acute administration of indole in the rat cecum caused a significant reduction in locomotion and an accumulation of indole metabolites in the brain, suggesting a possible direct role on central receptors. In contrast, chronic exposure to indole, achieved by the colonization of germ-free rats with *E. coli*, exacerbated anxiety-like and helplessness (i.e. depression-like) behaviors, but had no effect on motor activity [154]. In contrast to acutely administered animals, these colonized rats did not exhibit increased oxindole and isatin in the brain, nor increased circulatory corticosterone. These findings suggest that the behavioral alterations induced by chronic indole production (via colonization with indole-producing *E. coli*) are not mediated by the action of indole or its metabolites on central receptors or on the HPA axis [154]. A reduction in eye blinking frequency was detected, suggesting the involvement of the vagus nerve in eliciting the anxiogenic and depressive-like behaviors described [154].

**TABLE 4.** Studies investigating the effects of indole metabolites on depressive-like behavior.

**Treatment**	**Species or strain**	**Model**	**Behavioral outcomes**	**Molecular mechanisms**	**Reference**
Isatin (15 mg/kg i.p. in mice and 20 mg/kg i.p. in rats); yohimbine (2 mg/kg i.p. in mice and 2.5 mg/kg i.p. in rats)	Male Charles Foster rats and Wistar mice	-	↑anxiety in the OFT and EPM in mice, and the SIT in rats, comparable to yohimbine. ↓anxiolytic effects of diazepam in the OFT	-	[150]
Isatin (0–160 mg/kg i.p.)	Male Sprague-Dawley rats (90-100 days)	-	↑immobility in the OFT and FST	-	[151]
Oxindole or isatin (50 or 100 mg/kg, i.p.) or indole (500 mg/kg intra-cecal administration); inoculation with 1 mL of BW25113 or JW3686 bacterial cultures	F344 male rats (2–2.5 months)	Conventional, SPF and GF	Acute intra-cecal administration of indole induced ↓motor activity and ↑ concentrations of oxindole and isatin in the brain. Chronic overproduction of indole by colonization with *E. coli* caused no change in motor activity and no detectable oxindole or isatin in the brain but ↑ helplessness in the TST and ↑anxiety in the novelty test, EPM and OFT.	↑eye blinking frequency and ↑c-Fos protein expression in the dorsal vagal complex	[154]
Tryptophan-depleted diet with or without tryptophan supplementation; I3S, IPA, IAld or indole supplementation	female C57BL/6J mice (WT and GFAP AhR-deficient)	EAE	-	↑*Ccl2* and *Nos2* expression in astrocytes in tryptophan depleted group, reverted by supplementation; administration of I3S, IPA, IAld activates AhR and ↓*Ccl2* and *Nos2* expression	[153]

**AhR**: Aryl Hydrocarbon Receptor; **Ccl2**: C-C Motif Chemokine Ligand 2; **EAE**: Experimental Autoimmune Encephalomyelitis; **EPM**: Elevated Plus Maze; **FST**: Forced Swim Test; **GF**: Germ Free; **GFAP**: Glial Fibrillary Acidic Protein; **I3S**: Indoxyl-3-sulfate; **IAld**: Indole-3-aldehyde; **IPA**: Indole-3-propionic acid; **Nos2**: Nitric Oxide Synthase 2; **OFT**: Open Field Test; **SIT**: Social Interaction Test; **SPF**: Specific Pathogen Free; **WT**: Wild Type.

Indole and its derivatives (e.g. indoxyl-3-sulfate (I3S), indole-3-propionic acid (IPA) and indole-3-aldehyde (IAld)) are able to activate the aryl hydrocarbon receptor (AhR) [153, 155], with a subsequent inhibitory effect on neuroinflammation. Rothhammer *et al.* [153] showed in mice that were either supplemented with indole and related compounds or treated with tryptophanase, that neuroinflammation was reduced via activation of the AhR on astrocytes. This was attributed to increased expression of suppressor of cytokine signaling 2 (*Socs2*), and a subsequent inhibition of the transcription factor NF-kB.

Our understanding of the physiological and pathological role of indoles is hindered by the existence of a high number of indole derivatives, with diverse and dynamic actions. For example, IAld triggers the release of the anti-inflammatory cytokine IL-22 [156], IPA regulates intestinal barrier function via pregnane X receptor (PXR) [157] and is protective against DNA damage, lipid peroxidation and amyloid-β deposition in the brain [158, 159], and I3S is cytotoxic and triggers free radical production [160]. Additionally, there is a very small number of studies aimed at investigating the effect of these bioactive compounds on behavior. Given the tight link between tryptophan metabolism and mood, it is important to investigate the role of these molecules in order to understand the underlying mechanisms of this disease.

### Lactate

Lactate is an organic acid arising from both mammalian host processes and the fermentation of dietary fibers by lactic acid bacteria (e.g., *L. lactis, L. gasseri, and L. reuteri*), *Bifidobacteria* and *Proteobacteria* [161]. Lactate can be converted by several bacterial species to SCFAs contributing to the overall pool. Although present in the gut at low levels, lactate is absorbed into the bloodstream [162] and can cross the BBB [163]. Lactate has an established role in central signaling: in the brain, it is used as an energy substrate by neurons (due to its ability to be metabolized into glutamate) [164], it contributes to synaptic plasticity, and underlies memory formation [165, 166]. Both rodent and human studies support an association between depression and lactate abnormalities (**Table 5**). Increased concentrations of urinary lactate were measured in patients suffering from severe MDD compared to controls [167]. Interestingly, compared to conventionally colonized mice, germ-free mice exhibit elevated hippocampal concentrations of lactate, but decreased concentrations in the frontal cortex. In contrast, germ-free rats exhibit higher frontal concentrations of lactate than conventional rats [168].

**TABLE 5.** Studies investigating the effects of lactate on depressive-like behavior.

**Treatment**	**Species or strain**	**Model**	**Behavioral outcomes**	**Molecular mechanisms**	**Reference**
L-lactate (1 g/kg, ip, either acute or chronic (daily for 3 weeks))	C57Bl/6 mice (8-10 weeks)	corticosterone model of depression (for chronic experiment only)	Chronic treatment ↓ immobility in the FST to a similar extent as desipramine; chronic treatment abolished the increased immobility induced by corticosterone treatment in the FST and TST, reversed the corticosterone-induced decrease in saccharin consumption and decreased the immobility time in the open-space forced swim model of depression to a similar extent as desipramine	Acute effects: ↓ GSK3α and GSK3β in the hippocampus; ↓ phospho-CREB levels in the hippocampus; ↑hippocampal Arc, COX-2 and NOS1 mRNA expression; ↓COX-2 mRNA in the hippocampus. Chronic effects: ↑mRNA and protein levels encoding the regulator of serotonin receptors p11, the astrocytic marker S100β1 and the transcription factor Hes534 in the hippocampus of animals subjected to the open-space FST compared with vehicle-treated animals; ↓expression of PDE4D and NOS1 both at the mRNA and protein levels in the hippocampus of animals subjected to the open-space FST compared with vehicle-treated animals.	[174]
Lactate (during experimental stress period) or lactate + CI-994 (after experimental stress period)	male C57Bl/6 mice (8-10 weeks)	CSDS	Before the establihsment of depression: Reverses social avoidance and anxiety. After the establihsment of depression: reduced depression-like behavior	Before the establihsment of depression: Restores hippocampal class I HDAC2/3 levels and activity. After the establihsment of depression: hippocampal class II HDAC5 deactivation	[173]

**Arc**: Activity-Regulated Cytoskeleton-Associated Protein; **COX-2**: Cyclooxygenase 2; **CREB**: Camp Response Element-Binding Protein; **CSDS**: Chronic Social Defeat Stress; **FST**: Forced-Swim Test; **GSK3α/β**: Glycogen synthase kinase 3 alpha/beta; **HDAC2/3/5**: Histone Deacetylase 2/3/5; **NOS1**: Nitric Oxide Synthase 1; **PDE4D**: Camp-Specific 3′,5′-Cyclic Phosphodiesterase 4D; **TST**: Tail Suspention Test.

A potential mechanism through which lactate can modulate emotional behavior is through direct activation of the receptor GPR81 (also known as hydroxycarboxylic acid receptor 1 or HCA1), expressed in the hippocampus, neocortex and cerebellum [169]. The involvement of GPR81 in mood disorders has been suggested by Shoblock *et al.* [170]. However, through GPR81 activation, lactate modulates lipid and glucose metabolism, exerts an anti-inflammatory effect (also mediated by ARRB2) [171], and inhibits GABAergic neurotransmission [172].

An alternative, and significantly more explored, mechanism explaining the effect of lactate on depressive behavior is epigenetic regulation of depression-related genes. An interesting study by Karnib *et al.* (2019) revealed that lactate has both protective and reversing effects against depression, and that these processes occur via distinct epigenetic mechanisms on HDACs [173]. In this experiment, chronic lactate administration immediately before a 10-day social defeat challenge protected against the resulting social avoidance and anxiety behaviors observed in control mice. Lactate-treated mice exhibited increased levels and activity of the class I HDAC2/3 in the hippocampus [173]. In a second group of mice, which were not given lactate during the social stress challenge period, and that exhibited depressive-like symptoms, lactate had an antidepressant effect as shown by the rescue of social avoidance behavior. After the establishment of depression, the effect of lactate was not mediated by HDAC2/3; instead, it was mediated by a reduction in HDAC5 levels [173].

Carrard *et al.* (2018) also demonstrated the antidepressant effect of acute and chronic intraperitoneal injections of L-lactate in a corticosterone mouse model of depression. These behavioral effects followed an increase in the hippocampal concentrations of L-lactate, and were dependent on changes in the expression of several genes implicated in the pathophysiology of depression: GSK-α, GSK-β and CREB phosphorylation levels were significantly decreased, while the expression of Arc was increased and COX-2 and NOS1 decreased [174]. In addition to changes in the expression of depression-related or plasticity-related genes (GSK-α, GSK-β, CREB, Arc, COX-2 and NOS1), the behavioral effects of lactate were mediated by an increase in hippocampal p11 (regulator of 5-HT receptors), S100 β (astrocytic marker), Hes5 (transcription vector) and a decrease in cAMP-specific phosphodiesterase-4D (PDE4D) and NOS1 mRNA and protein levels [174].

Since lactate can also be synthetized by astrocytes on neuronal demand as a byproduct of glycolysis [175], it remains difficult to assess the net effect of microbial metabolism on central levels of lactate and mood. A simple way to isolate the contribution of the gut microbiome in the relationship between lactate production and depressive behavior would be using germ-free rodents; to the best of our knowledge, this has not been investigated to date. However, the well-established interchange of lactate between the periphery and the CNS [163] points towards a role of the gut microbiota in mediating the antidepressant effects of lactate. In support of this statement, the beneficial effects of exercise on mood have been hypothesized to be due to gut microbiota-mediated changes in the production of lactate [176, 177].

### Bile acids

Bile acids are cholesterol-derived steroid acids synthesized in the liver, secreted into the small intestine and absorbed in the ileum. The two primary bile acids (in humans and rats), cholic acid (CA) and chenodeoxycholic acid (CDCA), undergo further structural modifications in the gut by means of the gut microbiota, which convert them into secondary and tertiary bile acids [178]. Bile acids have local detergent properties that enables them to emulsify lipophilic molecules and, in turn, facilitate nutrient digestion and absorption. However, they can also act as signaling molecules to modulate feeding behavior and in turn, control glucose homeostasis, lipid metabolism and energy expenditure [179]. Their signaling pathways are initiated by their binding to the farnesoid X receptor (FXR) and the Takeda G protein-coupled receptor 5 (TGR5) [180].

The FXR is a nuclear receptor that is involved in the synthesis, secretion and transport of bile acids [181], as well as in the modulation of CREB activity [182]. Through its inhibitory control of the transcription factor CREB, bile acids can repress the transcription of several genes, including BDNF. Since the first reports of FXR expression in the brain [180, 183], the possibility has been explored that BDNF abnormalities found in the brains of depressed individuals may be accounted for, in part, by altered bile acid activity. Supportive of this hypothesis, the chronic unpredictable mild stress (CUMS) rodent model of depression exhibits enhanced hippocampal FXR expression, and in turn, FXR overexpression in the rat hippocampus is sufficient to induce depressive-like behavior in naïve animals [184]. These behavioral changes were mirrored by a significant decrease in BDNF expression in the hippocampus of rats overexpressing FXR. In contrast, FXR knockdown in naïve rats had a strong antidepressant effect as measured by the forced-swim and tail suspension tests, and prevented the occurrence of CUMS-associated behavioral (depressive-like symptoms) and molecular (decreased BDNF expression) abnormalities [184]. The antidepressant effect of FXR genetic deletion was confirmed in an independent study, which also reported altered glutamatergic, GABAergic, serotonergic, and noradrenergic neurotransmission in the hippocampus and cerebellum of FXR knockout mice, while no change was detected in the prefrontal cortex [185]. Deletion of FXR also led to disrupted bile acid metabolism and to increased bile acid abundance both peripherally and centrally [185, 186]. Different rodent models of depression have reported increased abundance of bile acids in urine and plasma [187], as well as in the fecal metabolic phenotype [188]. Su *et al.* [189], instead, reported an upregulation in serum glycocholic acid, but a decrease in cholic acid in chronic variable stress (CVS)-induced depression rats. These abnormalities were associated with a reduced abundance of *Peptostreptococcaceae incertaesedis* [188], supporting a link with altered microbiota function.

Moreover, bile acids may contribute to major depression by disrupting tight junction expression, leading to permeabilization of both intestinal and central epithelial cells [190]. Chenodeoxycholic acid or deoxycholic acid injections permealized the BBB in naïve rats [190]. When investigated in rat brain microvascular endothelial cells, increased BBB permeability upon administration of chenodeoxycholic acid or deoxycholic acid was found to be mediated by occludin phosphorylation in a Rac-1-dependent and FXR-independent fashion [190]. Enhanced permeabilization of intestinal epithelial barrier in human Caco-2 monolayers was associated with phosphorylation of the epithelial growth factor (EGF) receptor and dephosphorylation of the tight junction occludin. This occurred in response to administration of the hydrophobic bile acids cholic acid, chenodeoxycholic acid and deoxycholic acid, but not the hydrophilic bile acid ursodeoxycholic acid [191]. These findings suggest that the effect of bile acids may be to some extent dependent on their chemical and physical properties, which in turn, relies upon microbial-mediated modification of these compounds.

Another factor that may influence the behavioral outcome of bile acids is the receptor that mediates the response (**Table 6**). Binding of the TGR5 receptor by the secondary bile acid tauroursodeoxycholic acid (TUDCA) ameliorates the depressive phenotype of CUS mice by dampening neuroinflammation (TNF-α and IL-6), as well as oxido-nitrosative and endoplasmic reticulum stress [192]. This is consistent with previous reports of the neuroprotective effects of TUDCA in microglia [193]. Additionally, some bile acids, like lithocholic acid can stimulate central PXR and vitamin D receptor (VDR) [194], which have well-established antidepressant effects [195, 196]. Thus, the impact of bile acids on depressive behavior may be dependent on the specific receptor that they act upon, with FXR mediating pro-depressive phenotype, and PXR, VDR and TGR5 mediating their antidepressant action. This hypothesis has yet to be formally tested.

**TABLE 6.** Studies investigating the effects of bile acids on depressive-like behavior.

**Treatment**	**Species or strain**	**Model**	**Behavioral outcomes**	**Molecular mechanisms**	**Reference**
FXR knockout mice	C57BL/6 (4-5 months)	-	↓ immobility time in TST but not in FST (improved depressive-like symptoms); ↑ motor activity; impaired memory.	↓ hippocampal GAD65 and ↑ cerebral GAT1; changes in bile acid concentrations in serum (taurodehydrocholic acid, taurocholic acid, deoxycholic acid, glycocholic acid, tauro-α-muricholic acid, tauro-ω-muricholic acid, and hyodeoxycholic acid) and brain (taurocholic acid, taurodehydrocholic acid, tauro-ω-muricholic acid, tauro-β-muricholic acid, deoxycholic acid, and lithocholic acid)	[185]
FXR overexpression (LV-FXR-EGFP)	Male Sprague-Dawley rats (7 weeks)	-	Exacerbates depressive-like behavior in the FST, TST and SPT in naïve rats	No change in hippocampal expression of CREB and CRTC2; ↓ expression of BDNF in hippocampus.	[184]
FXR knockdown (LV-FXR-shRNA-EGFP)	Male Sprague-Dawley rats (7 weeks)	CUMS	Prevents depressive-like behavior in the FST, TST and SPT.	Restores decrease in hippocampal BDNF expression.	[184]
Chronic TUDCA (100, 200 mg/kg; ip) or fluoxetine (20 mg/kg; ip) or TUDCA + fluoxetine co-treatment for 10 days	Male C57BL/6J mice (8-10 weeks)	CUS	TUDCA (at 200 mg/kg) ↓ immobile time in TS and FST; ↑ crossing numbers in the OFT; ↑ sucrose intake in SPT compared to vehicle	TUDCA (at 200 mg/kg) ↓ TNFα and IL-6 in hippocampus and PFC	[192]

**BDNF**: Brain-Derived Neurotrophic Factor; **CREB**: Camp Response Element-Binding Protein; **CRTC2**: CREB-Regulated Transcription Coactivator 2; **CUMS**: Chronic Unpredictable Mild Stress; **CUS**: Chronic Unpredictable Stress; **FST**: Forced-Swim Test; **FXR**: Farnesoid X Receptor; **GAD65**: Glutamic Acid Decarboxylase 65; **GAT1**: GABA Transporter 1; **IL-6**: Interleukin-6; **OFT**: Open Field Test; **SPT**: Sucrose Preference Test; **Tnfα**: Tumor Necrosis Factor Alpha; **TST**: Tail Suspension Test; **TUDCA**: Tauroursodeoxycholic Acid.

### Choline metabolites

Choline is an essential nutrient mainly obtained from dietary lecithin and carnitine, but in humans, small amounts of choline can also be synthesized in the liver [197]. Choline has structural, epigenetic and cell signaling functions. It is involved in the synthesis of acetylcholine and it is a precursor of the cell membrane components phosphatidylcholine and sphingomyelin. Although not a bacterial product *per se*, choline is broken down by the action the gut microbiota into a range of metabolites, including trimethylglycine (betaine) and trimethylamine (TMA). In the liver, flavin monooxygenase, a family of xenobiotic-metabolizing enzymes, can further convert TMA into trimethylamine-*N*-oxide (TMAO) [198]. The role of the gut microbiota in choline metabolism is demonstrated by the positive association found between the plasma levels of TMA and TMAO with the microbial order *Clostridiales*, the genus *Ruminococcus*, and the taxon *Lachnospiraceae*, and the negative association with proportions of *S24-7*, an abundant family from *Bacteroidetes*, in mice [199]. In a CUMS rat model, depression was associated with increased TMA but decreased TMAO levels [200]. Since choline metabolism by the gut microbiota can deplete choline stores available for the host, excessive choline-utilizing bacteria can mimic the effects of choline deficiency, such as increased occurrence of metabolic diseases, higher cardiovascular risk, as well as altered behavior [201]. For example, reduced choline availability in the hippocampus and basal ganglia was reported in MDD patients [202, 203]. Reduced circulatory choline [117, 204], but elevated plasma TMAO [204] were also found in patients with depressive symptoms. However, this evidence is far from conclusive, as increased central concentrations of choline have been reported in depressed adults [195, 205, 206] as well as children and adolescents [207–209]. Moreover, the choline metabolites dimethylamine, dimethylglycine, and TMAO were found to be significantly lower in the urine of MDD subject compared to controls [210]. It is apparent that contradictory evidence exists with regards to the role of these microbial metabolites in the context of depression. The finding that urinary choline concentrations were lower in moderate MDD, but higher in severe MDD compared to matched control [211] hints to the complexity of choline metabolism in relation to depressive behavior.

Thus, different mechanisms may exist through which choline and its metabolites influence emotional behavior. One of these potential modes of action is DNA methylation. Romano *et al.* [201] showed that bacterial consumption of choline reduced the availability of methyl donors and altered global DNA methylation patterns in both the adult mice and their offspring, in line with previous reports of maternal choline deficiency inducing diminished hippocampal DNA methylation and neurodevelopmental abnormalities in the offspring [212]. Choline contributes to DNA methylation by modulating the production of the methyl donor S-adenosylmethionine (SAM) [201]. In a rat model of early-life stress, supplementation of choline and betaine and other methyl donors was successful in reversing depressive-like behavior [213]. In humans, betaine exhibited a positive effect on mood by promoting the DNA methylation of SAM: in subjects with mild MDD, adjunctive treatment of SAM with betaine showed higher antidepressant efficacy than treatment with SAM alone [214].

An alternative mechanism involves the modulation of neurotransmission. Oral ingestion of choline increases its concentrations in the brain [215], suggesting that dietary choline can contribute to acetylcholine synthesis. This suggests that abnormal choline metabolism may promote depressive behavior by altering the availability of choline destined for acetylcholine synthesis. In fact, the neurotransmitter acetylcholine is present in significantly higher concentrations in MDD patients than in healthy subjects [216]. Since choline can reach the CNS via active transport across the BBB [217], excessive choline in the periphery may have a significant impact on mood and behavior.

There remains uncertainty regarding the impact of choline metabolites on behavior (**Table 7**). While choline deficiency may be detrimental for mental health due to insufficient DNA methylation, excessive choline may contribute to depressive pathology by leading to enhanced acetylcholine synthesis. In addition, the extent to which the gut microbiota impacts on choline metabolism remains unknown, since clinical trials have shown that TMAO levels do not respond to prebiotic administration [218–220].

**TABLE 7.** Studies investigating the effects of choline metabolites on depressive-like behavior.

**Treatment**	**Species or strain**	**Model**	**Behavioral outcomes**	**Molecular mechanisms**	**Reference**
Methyl donor supplementation (choline, betaine, folate, vitamin B12) for 18 weeks	Wistar rats	ELS (maternal separation)	↓depressive behavior in the Porsolt FST	normalisation of total and HDL-cholesterol; ↑total DNA methylation and ↑hippocampal (not hypothalamic) expression of the insulin receptor	[213]

**HDL**: High-Density Lipoprotein; **FST**: Forced Swim Test.

### Vitamins (folate)

Most bacteria in the gut, such as *Lactobacillus* and *Bifidobacterium*, synthesize vitamins (particularly B-group vitamins and vitamin K) as part of their metabolic processes in the large intestine, and humans rely heavily on the gut microbiota for their production [221]. Vitamins are essential micronutrients with ubiquitous roles in a great number of physiological processes in several organs in the human body, including the brain. Fat-soluble vitamins (such as vitamins A, D, E, and K) make up the cell membrane, while water-soluble vitamins (including the vitamin B family and vitamin C) are enzymatic co-factors for a wide number of physiological reactions [221]. Active transporters are responsible for their transport across the BBB [222]. In the CNS, their role extends from energy homeostasis to neurotransmitter production [223], meaning that vitamin deficiencies can have a significant negative impact on neurological function (e.g. neural tube defects during fetal development). Folic acid, or vitamin B9, is a vitamin of microbial origin that has been extensively implicated in the pathology of depression (**Table 8**), with one third of depressed patients exhibiting a folate deficiency [224]. Its biosynthesis by the gut microbiota requires the C-N binding of 6-hydroxymethyl-7,8-dihydropterin pyrophosphate (DHPPP) – obtained from guanosine triphosphate (GTP) - and *p*-aminobenzoic acid (pABA) – a product of the pentose phosphate pathway [225].

**TABLE 8.** Studies investigating the effects of folate on depressive-like behavior.

**Treatment**	**Species or strain**	**Model**	**Behavioral outcomes**	**Molecular mechanisms**	**Reference**
Folic acid (75 mg/kg)	Male Sprague-Dawley rats	CUMS	Improvement of depression-like behaviors as assessed in FST, TST and OFT	↑5-HT, BDNF and GluR1 expression; changes in synaptic organisation in the brain	[227]
Folic acid (p.o. and i.c.v.) + PCPA (100 mg/kg) or fluoxetine (10 mg/kg, p.o.) or WAY100635 (0.1 mg/kg, s.c.) or ketanserin (5 mg/kg) or yohimbine (1 mg/kg, i.p.)	Male and female Swiss mice	-	↓immobility time in the FST; no effect on locomotor activity; PCPA blocked the decrease in immobility time elicited by folic acid; co-administration of a subeffective fluoxetine produced a synergistic effect with a subeffective dose of folic acid; WAY100635 significantly blocked the decrease in immobility time in the FST elicited by full dose of folic acid; WAY100635 produced a synergistic effect with a subeffective dose of folic acid; ketanserin blocked the decrease in immobility time in the FST elicited by folic acid; yohimbine was also able to prevent the anti-immobility effect the folic acid	-	[226]
folic acid (10 nmol/site, i.c.v.) + naloxone (1 mg/kg, i.p.) or naltrindole (3 mg/kg, i.p.) or naloxonazine (10 mg/kg, i.p.) or naloxone methiodide (1 mg/kg, s.c.)	Male and female Swiss mice	-	Naloxone, naltrindole, naloxonazine, but not naloxone methiodide, prevented the antidepressant-like effect of folic acid in the FST; folic acid + morphine had a synergistic anti-depressant effect in the FST (but no effect on locomotion); naloxone reversed the anti-depressant properties of folic acid + MK-801	-	[232]
Folate-depleted vs folate-supplemented diets	Adult male and female Wistar Kyoto rats	ELS (maternal separation)	dietary methyl donor supplementation induced ↑ exploratory behavior in the OFT, exhibit ↑social behavior and ↓ immobile time in the FST	↑DNA methylation in the hippocampus of mice exposed to maternal separation; ↑brain methionine levels in rats supplemented with methyl donors	[234]
Methyl donor supplementation (choline, betaine, folate, vitamin B12) for 18 weeks	Wistar rats	ELS (maternal separation)	↓depressive behavior in the Porsolt FST	normalisation of total and HDL-cholesterol; ↑total DNA methylation and ↑hippocampal (not hypothalamic) expression of the insulin receptor	[213]
Folic acid (5 or 10 nmol/i.c.v.; 25, 50 or 75 mg/kg p.o.), or fluoxetine (20 or 25 mg/kg) or 17-β estradiol (10 or 20 μg/rat); combination of folic acid (2.5 nmol/i.c.v.; or 25.0 mg/kg, p.o.) + fluoxetine (15.0 mg/kg); combination of folic acid (2.5 nmol/i.c.v.; or 25.0 mg/kg, p.o.) + 17-β estradiol (5 μg/rat)	Female Wistar rats	ovariectomized	↓ immobility in the FST; antidepressant effects were not achieved if ketanserin was admnistered.	-	[228]

**5-HT**: 5-Hydroxytryptamine; **BDNF**: Brain-Derived Neurotrophic Factor; **CUMS**: Chronic Unpredictable Mild Stress; **ELS**: Early-Life Stres; **FST**: Forced-Swim Test; **GluR1**: Glutamate Receptor 1; **HDL**: High-Density Lipoprotein; **MK-801**: Non-Competitive NMDA Receptor Antagonist; **OFT**: Open Field Test; **PCPA**: Para-Chlorophenylalanine; **TST**: Tail Suspension Test; **WAY100635**: 5-HT_1_A Receptor Antagonist And Full D_4_ Receptor Agonist.

Folate has an established antidepressant effect in animal models of depression [226–228], with some clinical studies suggesting its potential as antidepressant augmentation therapy in humans [229, 230]. Using a series of pharmacological inhibitors, Brocardo *et al.* [226] showed that the antidepressant effects of folic acid were dependent of serotonergic (5-HT_1A_ and 5-HT_2A/2C_ receptors) and noradrenergic (α1- and α2-adrenoceptors) activity in mice. The finding that serotonergic and noradrenergic antagonists prevented the antidepressant effects of folic acid supports the possibility that a mechanism of action is represented by an enhancement of monoaminergic production. Folic acid can synthesize tetrahydrobiopterin (BH4), which in turns act as a cofactor for the conversion of phenylalanine and tryptophan into the neurotransmitters dopamine, norepinephrine, and 5-HT [231]. With a similar design, the same group demonstrated that the antidepressant action of folic acid was mediated by the opioid system, as treatment of the mice with different opioid receptor antagonists prevented the folate-induced reduction in immobility time in the forced swim test [232]. The authors also proposed that the action of folic acid may involve inhibition of NMDA receptors [232].

In addition to increased central 5-HT concentrations, folic acid can also induce an increase in BDNF and GluR1 expression in the hippocampus and association cortex, concurrent with a normalization in serum corticosterone concentration, mitochondria structure and spine synapse numbers that were altered in the CUMS model of depression [227]. Due to its involvement in the synthesis of DNA, RNA and proteins and in DNA methylation reactions [233], folate may exert these changes via epigenetics mechanisms. A diet rich in methyl donors such as folic acid has beneficial effects on exploratory behavior, social interaction and depressive-like behavior in rats [213, 234]. The active metabolite of folate, 5-methyltetrahydrofolate (5-MTHF), converts homocysteine into methionine, which is used for the production of the methyl group donor SAM. In turn, SAM has been demonstrated to have antidepressant properties [235] via DNA methylation of phospholipids [236, 237], with extensive consequences on neurotransmission [238]. Despite the marked improvement in depressive behavior obtained in animal studies, clinical trials have highlighted great heterogeneity and do not provide strong evidence on the benefits of the use of folate as and adjunctive strategy for depression [239].

## FUTURE DIRECTIONS

Almost one third of depressed patients do not respond to treatment long-term [240]. The known impact of the microbiome on pathways involved in depression, as well as evidence linking abnormal microbiota and depressive behavior [14], suggest that targeting the gut microbiota may be an attractive strategy to improve depression-related pathological features. A strong advantage of this approach is the accessibility of the microbiome to nutritional modulation. In fact, administration of the probiotics *Bifidobacterium infantis, Lactobacillus rhamnosus, Lactobacillus helveticus R0052* and *Bifidobacterium longum R0175* have proved effective in normalizing the gut microbiome and alleviating anxiety- and depression-like symptoms in both rodents [55, 58] and healthy humans [241].

However, the complexity of the microbiota and its biochemical exchange with the host need to be better understood before this trans-kingdom communication can be harnessed to ameliorate neurological disorders. The contradictory findings reported across several studies may be reflective of this complexity. Components of the gut microbiota are in a dynamic state of equilibrium, dependent on substrate availability, exposure to antimicrobial compounds and competition with other bacterial strains. *In vitro*, the production of neuroactive metabolites by probiotics can be affected by nutrient availability [61]. Similarly, an intricate interplay exists between human and bacterial metabolism, as well as among the metabolic pathways reviewed. For example, intestinal neurotransmitter production is intrinsically linked to the abundance of SCFAs and bile acids in the gut, and inflammatory molecules like nitrate promote metabolism of choline by choline-utilizing bacteria [242], suggesting that the psychotropic effect of a specific metabolite may be tightly dependent on the presence of other metabolites. Another challenge encountered by gut-brain axis research is the ability to discriminate between peripheral production of neuroactive metabolites by the gut microbiota, and host production of the same metabolites in the brain. This makes it challenging to understand the extent to which the observed effect on depressive behavior can be ascribed to gut microbial metabolism *per se* (as compared to host central metabolism).

## CONCLUSION

MDD is a multifaceted mental disorder characterized by a dysfunction of neurochemical, neuroendocrine, immune and metabolic systems. The microbiota-gut-brain axis is a bidirectional network linking the central and enteric nervous systems through the same neural, immune and metabolic routes that are dysregulated in depression [243, 244]. Therefore, gut-brain axis abnormalities in depressed patients may, at least partly, account for the symptomatic presentations of depression. This review highlights how metabolites modulated by the intestinal microbiota can influence mood through their direct action on central receptors, through activation of peripheral receptors on neural, immune or neuroendocrine pathways, and through epigenetic regulation of histone deacetylation or DNA methylation (**Table 9**).

**TABLE 9.** Effects of microbial metabolites on depressive behavior in rodent and human studies.

**Microbial metabolite**	**Family**	**Metabolic pathway**	**Metabolising bacteria**	**Effects on helplessness (rodent studies)**	**Effects on mood (human studies)**	**Potential mechanisms**
Propionate	SCFA	Fermentation of fibres / carbohydrate metabolism	*Roseburia, Ruminococcus, Salmonella, Blautia, Phascolarctobacterium, Dialister, Coprococcus, Megasphaera*	Improves depressed mood	Depleted in MDD patients	Epigenetics (HDACi and DNA methylation modulator); receptors (GPR43, GPR41)
Acetate			*Blautia, Marvinbryantia*	-		
Butyrate			*Eubacterium, Roseburia, Anaerostipes, Coprococcus, Feacalibacterium*	Improves depressed mood; augments the effect of antidepressant drugs		
GABA	NT		*Lactobacillus, Bifidobacterium*	Antidepressant effect	Depleted in MDD patients	
Serotonin			*Escherichia coli, Streptococcus, Enterococcus, Akkermansia, Alistipes, Roseburia*	Antidepressant effect	Depleted in MDD patients	
Dopamine			*Escherichia coli, Bacillus cereus, Bacillus mycoides, Bacillus subtilis, Proteus vulgaris, Serratia marcescens and Staphylococcus aureus*	Antidepressant effect	Depleted in MDD patients	
Acetylcholine			*Lactobacillus*	-	Increased in MDD patients	
Oxindole	Indoles	Tryptophan metabolism	*Escherichia coli, Vibrio cholerae* and many others	Neurodepressant;	-	Epigenetics (HDACi); modulation of tryptophan availability; receptor AhR
Isatin				Anxiogenic and pro-depressive;	-	
Deoxycholic acid	Bile acids	Primary bile acid conjugation	*Lactobacillus, Enterococcus, Bacteroides, Clostridium*	permealization of the BBB	permealization of intestinal barrier (Caco-2 monolayer)	receptors (FXR and TGR5, PXR and VDR)
Glycocholic acid				increased in serum of depression model	-	receptors (FXR and TGR5, PXR and VDR)
TUDCA				neuroprotective against microglia	-	receptors (FXR and TGR5, PXR and VDR)
Taurocholic acid				FXR overexpression in the rat hippocampus is sufficient to induce depressive-like behavior, while FXR knockdown is both protective and reversing again depressive-like behavior; increased abundance of bile acids in urine, plasma and faecesof depression models	-	receptors (FXR and TGR5, PXR and VDR)
Betaine	Choline derivatives	Choline metabolism	*Bacteroidetes*	Reverses depressive-like behavior	Reduced in urine of MDD patients; ameliorates symptoms of depression	Affects abundance of choline available for DNA methylation and acetylcholine synthesis
TMA				-	Reduced in urine of MDD patients	
TMAO				-	Reduced in urine but elevated in plasma of MDD patients	
Lactate	-	Carbohydrate metabolism	*L. lactis, L. gasseri, and L. reuteri, Bifidobacteria and Proteobacteria, Eubacterium, Anaerostipes, Veillonella*	protective and reversing effects against depression	Increased in urine of MDD patients	Epigenetics (HDACi and DNA methylation modulator); receptor GPR81
Folate (B9)	Vitamin	GTP metabolism	*Lactobacillus (L. acidophilus, L. casei, L. paracasei, L. plantarum, L. reuteri, and L. salivarius) and Bifidobacterium*	antidepressant effect	enhances action of antidepressant drugs (but lack of conclusive evidence)	Epigenetics (DNA methylation modulator); serotonergic, noradrenergic, opioid and NMDA receptors; BH4 and SAM synthesis

**AhR**: Aryl Hydrocarbon Receptor; **BBB**: Blood Brain Barrier; **BH4**: Tetrahydrobiopterin; **FXR**: Farnesoid X Receptor; **GPR41**: G-protein coupled receptor 41; **GPR43**: G-protein coupled receptor 43; **GPR81**: G-protein coupled receptor 81; **GTP**: Guanosine triphosphate; **HDACi**: Histone deacetylase inhibitor; **MDD**: Major Depressive Disorder; **NT**: Neurotransmitter; **PXR**: Pregnane X receptor; **SAM**: S-adenosylmethionine; **SCFA**: Short-Chain Fatty Acids; **TGR5**: Takeda G-protein receptor 5; **TMA**: Trimethylamine; **TMAO**: Trimethylamine N-oxide; **TUDCA**: Tauroursodeoxycholic acids; **VDR**: Vitamin D Receptor

Addressing knowledge gaps on the multifactorial interplay between products of microbial metabolism in relation to their antidepressant effects will advance our understanding of the pathological mechanisms of depression (via the gut-brain axis) and may facilitate the development of more refined pharmacological strategies.

## Abbreviations:

3-HK: – 3-hydroxykynurenine,
5-HIAA: – 5-hydroxyindole acetic acid,
5-HT: – 5-hydroxytryptamine,
AhR: – aryl hydrocarbon receptor,
BBB: – blood-brain barrier,
BDNF: – brain-derived neurotrophic factor,
CNS: – central nervous system,
CUMS: – chronic unpredictable mild stress,
FXR: – farnesoid X receptor,
GABA: – γ-aminobutyric acid,
GI: – gastrointestinal,
HDAC: – histone deacetylase,
HPA: – hypothalamic pituitary adrenal,
IAld: – Indole-3-aldehyde,
IDO: – Indoleamine-2,3-dioxygenase,
IFN: – interferon,
IL: – interleukin,
IPA: – indole-3-propionic acid,
KYNA: – kynurenic acid,
LPS: – lipopolysaccharide,
MDD: – major depressive disorder,
NMDA: – N-methyl-D-aspartate,
PXR: – pregnane X receptor,
SAM: – S-adenosylmethionine,
SCFA: – short-chain fatty acid,
TDO: – tryptophan-2,3-dioxygenase,
TET: – ten-eleven translocation,
TGR5: – Takeda G protein-coupled receptor 5,
TMA: – trimethylamine,
TMAO: – trimethylamine-N-oxide,
TNF-α: – tumor necrosis factor-α,
TPH: – tryptophan hydroxylase,
TUDCA: – tauroursodeoxycholic acid,
VDR: – vitamin D receptor.
